# Evaluation of unfractionated heparin therapy for venous thromboembolism using adjusted body weight in elderly or higher weight patients

**DOI:** 10.1007/s11239-024-03060-4

**Published:** 2024-12-04

**Authors:** Arielle J. Hopkins, Terence Chau, Benjamin Pullinger, Sungwook Kim, Justin J. Delic, Lauren A. Igneri, Soyoung Kim

**Affiliations:** 1 Ernest Mario School of Pharmacy, Rutgers University, New Brunswick, NJ USA; 2 Cooper University Hospital, One Cooper Plaza, Camden, NJ 08103 USA; 3 Philadelphia College of Pharmacy, Saint Joseph’s University, Philadelphia, PA USA; 4 Saint Joseph’s University, Philadelphia, PA USA

**Keywords:** Heparin, Anticoagulation, Weight-based, Anti-Xa, Therapeutic drug monitoring, Venous thromboembolism

## Abstract

The use of weight-based unfractionated heparin (UFH) infusions is the standard of care in hospital management of venous thromboembolism (VTE). Initial dosing strategies for UFH in older adults and higher body weight patients remain uncertain given differences in pharmacokinetics and concerns for over-anticoagulation. Methods: This was a single-center, retrospective, pre-post study involving older adults aged ≥ 65 years and patients weighing ≥ 100 kg with suspected or confirmed VTE to determine if the use of adjusted body weight (AdjBW)-based UFH regimens improves time to therapeutic anti-Xa levels compared to total body weight (TBW)-based regimens Patients received weight-based UFH infusions, dosed according to either TBW or AdjBW, to target a therapeutic anti-Xa level. Each cohort consisted of 40 patients, stratified by whether they met age or weight criteria to ensure equal representation of elderly and higher body weight patients between cohorts. The median time to therapeutic anti-Xa levels was shorter in the AdjBW group compared to the TBW group (13.6 h versus 20.9 h; point estimate 5.3 h (95% CI 0.2 to 9.9)). This finding was driven by those aged ≥ 65 years and those who received a bolus dose at the start of the infusion. Among older adults and higher weight adults with suspected or confirmed VTE, the use of AdjBW to guide heparin infusion initiation was associated with shorter time to therapeutic anti-Xa levels. This finding driven by the older adult sample and the subgroup analyses did not find a statistically significant difference in time to therapeutic anti-Xa levels in higher body weight patients aged less than 65 years.

**Graphical abstract:**

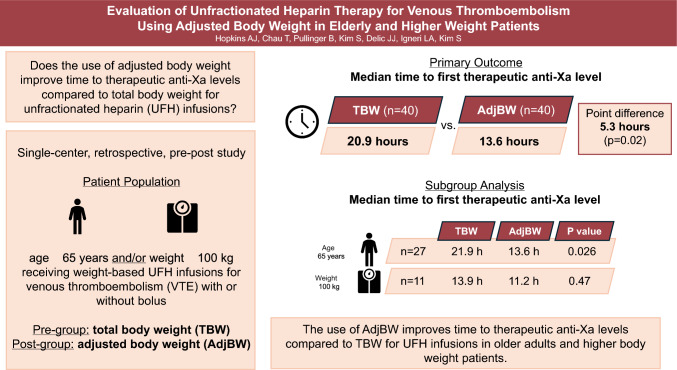

**Supplementary Information:**

The online version contains supplementary material available at 10.1007/s11239-024-03060-4.

## Highlights

- Optimal initial dosing strategies for UFH infusion in older adults and higher weight patients remain uncertain.
- This was a single-center, retrospective, pre-post study involving older adults and higher weight patients with suspected or confirmed VTE to determine if the use of AdjBW-based UFH regimens improves time to therapeutic anti-Xa levels compared to TBW-based regimens.
- The use of AdjBW to guide heparin infusion initiation was associated with shorter time to therapeutic anti-Xa levels.
- Larger, prospective studies are required to determine impact on clinical safety and efficacy outcomes.

## Background

Unfractionated heparin (UFH) is a parenteral anticoagulant utilized as standard of care in the treatment of venous thromboembolism (VTE), including deep vein thrombosis (DVT) and pulmonary embolism (PE), either alone or in combination with fibrinolytic therapy [1]. Its short half-life makes it an ideal anticoagulant when there is a risk of bleeding, or when thrombolysis or other procedures may be indicated [2]. Frequent monitoring of UFH in the inpatient setting is made possible using lab values such as the anti-factor Xa (anti-Xa) assay, which indirectly measures the amount of residual factor Xa to extrapolate a presumed UFH concentration, or activated partial thromboplastin time (aPTT), which serves as a reflection of the intrinsic and common pathways of the coagulation cascade [3]. Standardized, weight-based protocols for the initial dosing and titration of UFH is the standard of care in many medical centers with the intention of rapidly achieving therapeutic anticoagulation [4]. However, a previous study showed that higher weight individuals (≥ 100 kg) require a higher total dose (in units/hour), but a lower weight-based dose (in units/kg/hr), to achieve therapeutic anticoagulation compared with lower weight patients (< 100 kg) [5]. Adipose tissue has disproportionately less blood volume than lean tissue, causing a decrease in overall volume of distribution of UFH and therefore lower weight-based UFH requirements as weight increases [5].

Similarly, over-anticoagulation may occur in older adults given the potential for changes in intrinsic clotting factor concentrations and renal clearance of heparin [6–9]. One study further hypothesized that as patients age, the volume of distribution of heparin likely decreases in the setting of increased adipose tissue and decreased muscle tissue [10]. Barletta et al. identified increasing age as an independent risk factor for supratherapeutic aPTT values [11]. Over-anticoagulation with UFH may increase the risk of bleeding, thus creating a need to determine the optimal dosing strategy for this population.

The use of adjusted body weight (AdjBW) in pharmacokinetic models for higher weight patients has been established in previous studies [5, 11–13]. Guervil et al. evaluated an approach in which patients weighing greater than 125 kg were administered UFH based on AdjBW, but no direct comparison of TBW versus AdjBW dosing strategies was performed [13]. Another study found faster times to therapeutic aPTT when comparing AdjBW versus TBW in patients weighing over 100 kg [14]. However, this study did not confirm the hypothesis using the anti-Xa assay, nor did it study any difference between older and younger adults.

In 2019, our medical center adjusted its TBW-based UFH protocol to utilize AdjBW in adults 65 years or older or weighing greater than or equal to 100 kg. The purpose of this retrospective study was to compare the safety and efficacy of the previous TBW-based protocol to the AdjBW-based protocol in these populations. It was predicted that utilizing the patients’ AdjBW would shorten the time to first therapeutic anti-Xa value.

## Methods

### Design and patients

This single-center, retrospective, pre-post cohort study included adult patients admitted with suspected or confirmed VTE who received UFH infusion through a standardized protocol for at least 24 h without interruption of the infusion, other than when the infusion was held according to the protocol. Based on the institutional VTE protocol, UFH infusions were initiated at a rate of 18 units/kg/hr with or without initial and adjustment bolus doses per provider discretion. Patients’ anti-Xa assays were collected six hours after initiating the infusion, and infusion rates were adjusted in a weight-based fashion in order to target a therapeutic anti-Xa goal of 0.3 to 0.7 units/mL. The titration protocol, including bolus doses, can be found in Online Resource 1. The anti-Xa assay was checked every six hours until two consecutive therapeutic anti-Xa levels were obtained, at which point the assay was checked every 24 h until the infusion was discontinued or a non-therapeutic anti-Xa level was obtained. Patients admitted to Cooper University Hospital during the following time periods were screened for eligibility: July 1, 2019 through September 30, 2019 (TBW cohort) and July 1, 2021 through September 30, 2021 (AdjBW cohort).

Patients were included if they were ≥ 18 years old, had at least one anti-Xa level six hours after initiation of heparin, and met at least one of the two criteria evaluated: weight ≥ 100 kg and/or age ≥ 65 years old.

Patients were excluded if there was any significant variation from the approved institution-based heparin protocol, if they were pregnant or actively incarcerated, received an oral factor Xa inhibitor during or directly prior to the index admission before UFH was initiated, or underwent extracorporeal membrane oxygenation during the UFH infusion. Significant variation from the protocol included an anti-Xa level collected earlier than five hours following the most recent change in infusion rate, any change to infusion rate not in accordance with the protocol, incorrect weight used, initiation rate different from 18 units/kg/hr, and initial bolus dose significantly different from 80 units/kg. Additionally, patients with missing height or height ≤ 152.4 cm were excluded. This study was approved by the local institutional review boards.

### Endpoints

The primary endpoint was the median time to first therapeutic anti-Xa level of 0.3 to 0.7 units/mL following initiation of the heparin infusion. Secondary endpoints included percentage of first-measured anti-Xa values in the therapeutic range and percentage of therapeutic anti-Xa values within the first 24 h of starting the UFH infusion. Safety endpoints included inpatient mortality and major bleeding, defined by the International Society of Thrombosis and Haemostasis (ISTH) as overt bleeding associated with a decrease in hemoglobin level of ≥ 2 g/dL, requiring a blood transfusion of at least two units of whole blood or red cells, occurring in a critical site, or contributing to death [15].

### Statistical analysis

The time frames studied were chosen to avoid the early COVID-19 era, when virus-induced coagulopathy was a poorly understood concern. This limits the generalizability of our study to patients with COVID-19-induced coagulopathy who may have different heparin requirements. The primary endpoint was analyzed with the Mann Whitney U-test, which was selected for comparing the true population medians. The Hodges Lehmann estimator was used to estimate the difference between the population medians. The secondary endpoints were analyzed using Pearson’s Chi Square test or with the Fisher Exact test for low frequency results. Patients were stratified and analyzed in the following subgroups: (1) patients weighing ≥ 100 kg, (2) patients aged ≥ 65 years, and (3) patients weighing ≥ 100 kg and aged ≥ 65 years. A post-hoc subgroup analysis was also conducted to determine any difference detected between those patients who did versus did not receive an 80 units/kg bolus dose at the start of the heparin infusion. All subgroups were analyzed using the same statistical methods as the primary endpoint. Statistical analyses were performed using R statistical software (version 4.3.0).

## Results

**Fig. 1:**
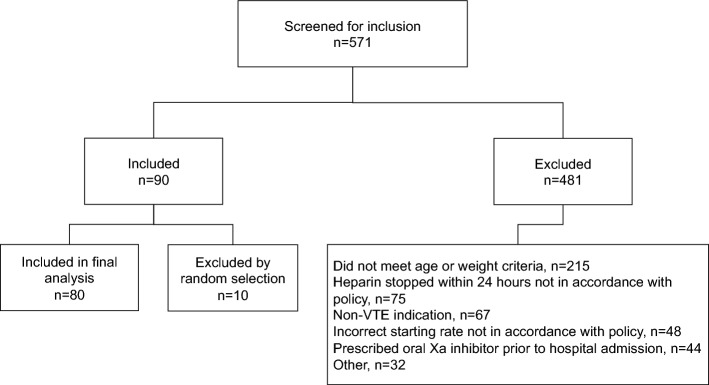
Enrollment

Five hundred seventy-one patients were screened for inclusion, and 90 patients met all inclusion and no exclusion criteria (Fig. 1). To ensure the same number of patients in the elderly and high body weight strata, 10 patients were randomly excluded, leaving 40 patients in each cohort (total of 80 patients). The baseline characteristics are summarized in Table 1. Twenty-seven (67.5%) patients in each group were aged ≥ 65 years old and weighed < 100 kg, eleven (27.5%) patients in each group weighed ≥ 100 kg but were aged < 65 years old, and two (5%) patients in each group met both criteria for AdjBW-based heparin dosing. Thirteen (32.5%) patients in each cohort received a bolus dose of 80 units/kg at the start of the UFH infusion. The mean age of included patients was 68.8 years old in the TBW cohort and 67.3 years old in the AdjBW cohort. The mean weight was 92 kg in the TBW cohort and 85 kg in the AdjBW cohort.

**Table 1:** Baseline characteristics

	TBW Cohort, n = 40	AdjBW Cohort, n = 40	P values
Age in years—mean (SD)	68.8 (16.69)	67.3 (11.82)	0.64
TBW in kg—mean (SD)	91.02 (33.68)	84.76 (22.86)	0.33
Age ≥ 65 years only—no. (%)	27 (67.5)	27 (67.5)	1
Weight ≥ 100 kg only—no. (%)	11 (27.5)	11 (27.5)	1
Age ≥ 65 years and weight ≥ 100 kg—no. (%)	2 (5)	2 (5)	1
Received bolus dose of 80 units/kg—no. (%)	13 (32.5)	13 (32.5)	1
Male sex—no. (%)	19 (47.5)	20 (50)	0.82
Race, White (non-Hispanic)—no. (%)	25 (62.5)	27 (67.5)	0.64
Race, Hispanic—no. (%)	2 (5)	3 (7.5)	0.64
Race, African American—no. (%)	13 (32.5)	8 (20)	0.2
Race, Other—no. (%)	0 (0)	2 (5)	0.17
Location, Med/Surg Unit—no. (%)	29 (72.5)	27 (67.5)	0.63
Location, Intensive Care Unit—no. (%)	11 (27.5)	13 (32.5)	0.63
Concomitant antithrombotic medication used—no. (%)^a^	25 (62.5)	34 (85)	0.02
Concomitant alteplase used—no. (%)	2 (5)	4 (10)	0.39
Serum creatinine—mean (SD)	1.24 (0.80)	1.56 (2.32)	0.42

*No*. number, *SD* standard deviation

^a^Aspirin, clopidogrel, ticagrelor, warfarin

The primary and secondary outcomes are summarized in Table 2. The median time to first therapeutic anti-Xa level was significantly shorter in the AdjBW cohort compared with the TBW cohort (13.6 versus 20.9 h; point estimate 5.3 h; 95% confidence interval [CI] 0.2–9.9; p = 0.02). This difference was driven by the age subgroup (point estimate 7.0 h; 95% CI 0.2 to 12.9; p = 0.026) as well as by patients who received a bolus dose at the start of the infusion (point estimate 8.6 h; 95% CI 1.2 to 14.6; p = 0.02) (Table 2). There were no statistically significant differences detected in the weight (p = 0.47) or no initial bolus dose (p = 0.21) subgroups, although the times to goal were numerically shorter in the AdjBW cohort. The subgroup analysis was not performed on patients who met both age and weight criteria due to low sample size.

**Table 2:** Primary and secondary outcomes

	TBW Cohort, n = 40	AdjBW Cohort, n = 40	Point Estimate* [95% confidence interval]	P value
Median time to first therapeutic anti-Xa, hours (IQR)				
Total cohort; n = 40 in each cohort	20.9 (6.7–27.7)	13.6 (6.5–18.9)	5.3 [0.2–9.9]	0.02
Age ≥ 65 years only; n = 27 in each cohort	21.9 (10.9–28.0)	13.6 (6.2–19.4)	7.0 [0.2–12.9]	0.026
Weight ≥ 100 kg only; n = 11 in each cohort	13.9 (6.7–25.9)	11.2 (6.4–16.5)	2.7 [−5.0–12.4]	0.47
Bolus at start of infusion; n = 13 in each cohort	22.7 (17.4–27.6)	13.7 (12.3–15.0)	8.6 [1.2–14.6]	0.02
No bolus at start of infusion; n = 27 in each cohort	20.9 (6.5–27.1)	13.5 (6.3–21.7)	0.8 [-0.6–9.3]	0.21
First-measured anti-Xa within therapeutic range—no. (%)	12 (30)	17 (43)	–	0.24
Therapeutic anti-Xa values within the first 24 h of IV UFH protocol—no./total n(%)	40/103 (38.8)	59/105 (56.2)	–	0.01
In-hospital mortality—no. (%)	3 (7.5)	4 (10)	–	1
Major bleeds—no. (%)	4 (10)	2 (5)	–	0.68
Heparin dose at therapeutic anti-Xa				
In units/hr—mean (SD)	1389 (594)	1302 (400)	–	–
In units/kg TBW/hr—mean (SD)	15 (4.0)	16 (3.5)	–	–

*Using the Hodges-Lehmann estimate; calculated for continuous outcomes

*IQR* interquartile range, *No.* number, *SD* standard deviation

**Fig. 2:**
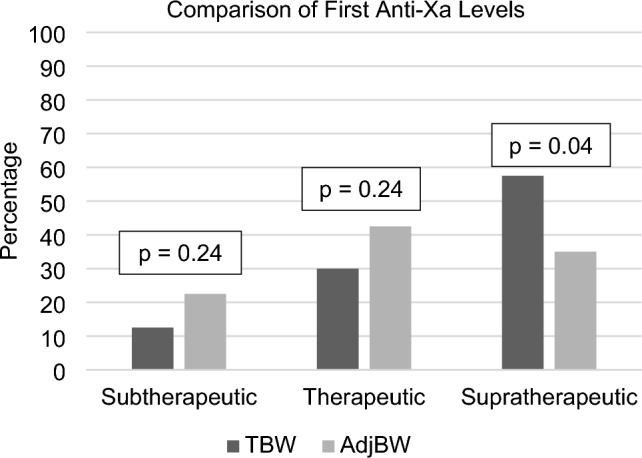
Comparisons of first collected anti-Xa level using Chi Square tests

The AdjBW cohort had a higher percentage of therapeutic anti-Xa values within the first 24 h versus the TBW cohort (53% in the AdjBW cohort versus 40% in the TBW cohort; p = 0.02), but no statistically significant difference was observed in the percentage of first-measured anti-Xa values in the therapeutic range (42.5% versus 30%; p = 0.24). The breakdown of the first measured anti-Xa levels is illustrated in Fig. 2. A lower rate of first anti-Xa levels were supratherapeutic with the use of AdjBW (p = 0.04); however, this did not result in a significant increase in the rate of subtherapeutic levels (p = 0.24).

There were no statistical differences in the rates of inpatient mortality between the TBW and AdjBW groups (7.5% versus 10%, p = 1) as well as major bleeds (10% versus 5%, p = 0.68). Descriptions of each major bleeding event are summarized in Online Resource 2.

## Discussion

In this study, we compared the use of AdjBW versus TBW-based heparin dosing in older adults and higher weight patients. The median time to therapeutic anti-Xa level was significantly shorter in the AdjBW cohort compared with the TBW cohort. In subgroup analyses, this improvement in time to therapeutic anti-Xa was only observed in patients ≥ 65 years old. To our knowledge, this is the first study that directly compares the effects of AdjBW versus TBW dosing on anti-Xa outcomes in older adults [6–9]. The results of our study support the hypotheses generated by Schurr et al. and Barletta et al. who both found evidence supporting the need for an adjustment to heparin dosing that takes advanced age into account [10, 11]. Schurr et al. also demonstrated that utilizing AdjBW in higher weight and older adult patients significantly reduces the risk of the first collected anti-Xa value being supratherapeutic, which was supported by our results as well.

In terms of the weight subgroup, it is important to note that only eleven patients in each group exclusively met the weight criteria, and this analysis was likely underpowered to detect a statistically significant difference. The benefits of utilizing AdjBW for higher weight patients have been previously studied [5, 11, 13]. When George et al. divided patients into weight-based cohorts (< 100 kg, 100–124.9 kg, 125–150 kg, and > 150 kg), they found that higher body weight patients (≥ 100 kg) required a higher unit/hr dose, but a lower weight-based unit/kg/hr dose compared with lower body weight patients (< 100 kg) in order to achieve therapeutic aPTT levels. Based on this previously published data, the use of AdjBW in higher weight patients is reasonable even if they do not meet the age criteria.

Our study also found that the use of AdjBW improved the time to therapeutic anti-Xa levels specifically in patients who received an initial 80 unit/kg bolus dose. Given that bolus doses are administered to achieve rapid therapeutic anticoagulation in the presence of an active thrombus, our findings support the use of AdjBW for weight-based boluses. This may minimize the risk of bleeding while continuing to provide adequate anticoagulation in the setting of thromboembolism.

There were several instances of major bleeding events in this study (10% and 5% in the TBW and AdjBW cohorts, respectively). Of the four bleeding events noted in the TBW cohort, two received Naranjo scores of greater than 4, indicating that the adverse event was possibly due to the use of UFH (Online Resource 2). The two bleeding events noted in the AdjBW cohort received low Naranjo scores due to the lack of temporal association and presence of alternative causes.

This study is limited by its retrospective, single-center nature. Many hospitals utilize an institution-specific protocol for monitoring and titrating UFH infusions, which limits the generalizability of this study. There were two factors noted that may have contributed to lengthened times to therapeutic anti-Xa levels as well. First, anti-Xa levels drawn greater than six hours after infusion initiation or a rate change were included in the analysis. There were two patients in each cohort whose first anti-Xa levels were collected over 10 h following UFH initiation. Second, several blood samples were reported as hemolyzed, requiring the samples to be redrawn. While this issue impacted similar amounts of patients in the TBW (12 of 249 samples) and AdjBW cohorts (10 of 259 samples), the hemolyzed samples may have delayed time to therapeutic anti-Xa by several hours for patients that had not yet achieved a therapeutic anti-Xa, five patients in the TBW group and three patients in the AdjBW group. Additionally, the primary outcome of this study focused on laboratory values rather than patient-centered endpoints such as successful VTE treatment or decrease in bleeding rates. Lastly, the time frames studied were chosen to avoid the early COVID-19 era, when virus-induced coagulopathy was a poorly understood concern. This limits the generalizability of our study to patients with COVID-19-induced coagulopathy who may have different heparin requirements. The promising findings from this study warrant a larger, prospective randomized controlled trial to strengthen the hypothesis that utilizing AdjBW-based dosing is efficacious in treating VTE and will minimize the risk of bleeding compared to TBW-based dosing.

To obtain the most accurate anti-Xa assessments, 44 patients were excluded for use of oral factor-Xa inhibitors, such as apixaban or rivaroxaban, during or directly prior to the index admission. Given the increasing use of these medications for prior VTE and stroke prophylaxis in patients with atrial fibrillation, additional studies should be conducted in these populations to assess clinical outcomes.

Some strengths of this study include its real-life applicability given the inclusion of inconclusive anti-Xa assays and allowance for minor delays in anti-Xa testing. Additionally, the use of anti-Xa in this study allowed monitoring of UFH infusions that was not impacted by the variables that impact aPTT monitoring, such as liver dysfunction, hypercoagulable states, and abnormal Factor II and VIII concentrations. The time periods selected for this study also carefully avoided the early COVID-19 era, during which COVID-19-induced coagulopathy was of greater concern and not as well understood. Finally, by stratifying patients during the inclusion process, it was ensured that the sample evaluated was balanced in terms of weight and age.

In conclusion, the results of our study find that the use of AdjBW-based dosing of UFH improves time to therapeutic anti-Xa levels in older adults and higher body weight. This finding was seen primarily in older adults and the subgroup analyses did not find a statistically significant difference in time to therapeutic anti-Xa levels in higher body weight patients who did not meet the older age criteria.

## Supplementary Information

Below is the link to the electronic supplementary material.

Supplementary file1 (DOCX 16 KB)

Supplementary file2 (DOCX 15 KB)

## Data Availability

The data that support the findings of this study are not publicly available due to the sensitive nature of the research involving human participants and the need to protect their privacy. De-identified data may be accessed upon reasonable request by contacting the corresponding author, subject to approval by the Institutional Review Board (IRB) and adherence to strict data usage guidelines.
