# Supplementary material for: Evaluation of unfractionated heparin therapy for venous thromboembolism using adjusted body weight in elderly or higher weight patients
Source: J Thromb Thrombolysis. 2024 Dec 4;58(3):420–6. doi: 10.1007/s11239-024-03060-4 (PMC12009232; doi:10.1007/s11239-024-03060-4)
Supplement: Supplementary file 1 — Supplementary file1 (DOCX 16 KB) [file 11239_2024_3060_MOESM1_ESM.docx]

Supplemental Table 1: UFH Protocol

| **Heparin, Unfractionated Level (units/mL)** | **Bolus Dose** | **Heparin dose adjustment** | **Repeat Heparin, Unfractionated Level after rate change** |
| --- | --- | --- | --- |
| Less than 0.15 | 80 units/kg | Increase by 4 units/kg/hr | 6 hours |
| 0.15-0.29 | 40 units/kg | Increase by 2 units/kg/hr | 6 hours |
| 0.30-0.70 | None | None | 6 hours (once two consecutive unfractionated heparin levels within range, repeat every 24 hours) |
| 0.71-0.85 | None | Decrease by 2 units/kg/hr | 6 hours |
| Greater than 0.85 | None | Hold infusion for 1 hour, then decrease by 3 units/kg/hr | 6 hours |

Evaluation of Unfractionated Heparin Therapy for Venous Thromboembolism Using Adjusted Body Weight in Elderly and Higher Weight Patients

Journal of Thrombosis and Thrombolysis

Arielle J. Hopkins, PharmD, BCPS^1^; Terence Chau, PharmD, BCPS, BCCCP, BCEMP^2^; Benjamin Pullinger, PharmD, BCPS^3^; Sungwook (Peter) Kim, PhD^4^; Justin J. Delic, PharmD, BCCCP^2^; Lauren A. Igneri, PharmD, BCPS, BCCCP, FCCM^2^; Soyoung (Kristi) Kim, PharmD, BCCCP^2^

^1^ Ernest Mario School of Pharmacy, Rutgers University, New Brunswick, New Jersey

^2^ Cooper University Hospital, Camden, New Jersey

^3^ Saint Joseph’s University, Philadelphia, Pennsylvania

^4^ Philadelphia College of Pharmacy, Saint Joseph’s University, Philadelphia, Pennsylvania

Corresponding author’s email address: arielle.j.hopkins@gmail.com
