# Supplementary material for: Evaluation of unfractionated heparin therapy for venous thromboembolism using adjusted body weight in elderly or higher weight patients
Source: J Thromb Thrombolysis. 2024 Dec 4;58(3):420–6. doi: 10.1007/s11239-024-03060-4 (PMC12009232; doi:10.1007/s11239-024-03060-4)
Supplement: Supplementary file 2 — Supplementary file2 (DOCX 15 KB) [file 11239_2024_3060_MOESM2_ESM.docx]

Supplemental Table 2: Description of Bleeding events

| **Age (years)** | **TBW (kg)** | **Cohort** | **Brief Description of Bleeding Event** | **Average recorded anti-Xa level (unit/mL)** | **Highest anti-Xa level recorded prior to onset of bleed (unit/mL)** | **Time to first therapeutic anti-Xa (hours)** | **Naranjo Score** |
| --- | --- | --- | --- | --- | --- | --- | --- |
| 67 | 59 | TBW | Arterial bleeding at sheath site during thrombolysis and retroperitoneal hematoma. Hemoglobin dropped by approximately 4 g/dL and required transfusion of packed red blood cells | 0.49 | 0.53 | 7.43 | 3 |
| 65 | 51 | TBW | Recurrent upper gastrointestinal bleed requiring endoscopic intervention and blood transfusions | 0.73 | 0.88 | 14.30 | 7 |
| 45 | 103 | TBW | Bleed following laparoscopic bowel surgery. Two units of packed red blood cells were transfused along with fresh frozen plasma | 0.86 | 1.28 | 6.90 | 4 |
| 78 | 72 | TBW | Found to have two areas of intracranial hemorrhage on computed tomography | 0.70 | 1.45 | 23.12 | 8 |
| 65 | 74 | AdjBW | Hemoglobin dropped by 2 g/dL in a post-surgical patient, requiring transfusion of red blood cells on post-op day 2. Event occurred again on post-op day 6, requiring additional red blood cell transfusion | 0.48 | 0.88 | 17.35 | 2 |
| 46 | 102 | AdjBW | Hemoglobin dropped by 4 g/dL in a patient presenting with a gunshot wound, initially requiring 4 units of packed red blood cells to be transfused. | 0.47 | 0.64 | 6.28 | 0 |

Evaluation of Unfractionated Heparin Therapy for Venous Thromboembolism Using Adjusted Body Weight in Elderly and Higher Weight Patients

Journal of Thrombosis and Thrombolysis

Arielle J. Hopkins, PharmD, BCPS^1^; Terence Chau, PharmD, BCPS, BCCCP, BCEMP^2^; Benjamin Pullinger, PharmD, BCPS^3^; Sungwook (Peter) Kim, PhD^4^; Justin J. Delic, PharmD, BCCCP^2^; Lauren A. Igneri, PharmD, BCPS, BCCCP, FCCM^2^; Soyoung (Kristi) Kim, PharmD, BCCCP^2^

^1^ Ernest Mario School of Pharmacy, Rutgers University, New Brunswick, New Jersey

^2^ Cooper University Hospital, Camden, New Jersey

^3^ Saint Joseph’s University, Philadelphia, Pennsylvania

^4^ Philadelphia College of Pharmacy, Saint Joseph’s University, Philadelphia, Pennsylvania

Corresponding author’s email address: arielle.j.hopkins@gmail.com
